# The effects of protease, xylanase, and xylo-oligosaccharides on growth performance, nutrient utilization, short-chain fatty acids, and microbiota in *Eimeria*-challenged broiler chickens fed low-protein diet

**DOI:** 10.1016/j.psj.2023.102789

**Published:** 2023-05-19

**Authors:** Yang Lin, Jeferson M. Lourenco, Oluyinka A. Olukosi

**Affiliations:** ⁎ Department of Poultry Science, University of Georgia, Athens, GA, USA; † Department of Animal and Dairy Science, University of Georgia, Athens, GA, USA

**Keywords:** *Eimeria*, xylanase, protease, xylo-oligosaccharides, microbiota

## Abstract

A total of 392 Cobb 500 off-sex male broiler chicks were used in a 21-day experiment to study the effect of protease, xylanase, and xylo-oligosaccharides (**XOS**) on improving growth performance, nutrient utilization (ileal digestibility and total tract retention), gene expression of nutrient transporters, cecal short-chain fatty acids (**SCFAs**), and microbiota profile of broilers challenged with *Eimeria* spp. Chicks at 0-day old were allocated to 8 treatments in a 4 × 2 factorial arrangement: 1) corn-soybean meal diet with no enzyme (**Con**); 2) Con plus 0.2 g/kg protease alone (**PRO**); 3) Con plus 0.2 g/kg protease combined with 0.1 g/kg xylanase (**PRO + XYL**); or 4) Con plus 0.5 g/kg xylo-oligosaccharides (**XOS**); with or without *Eimeria* challenge. The 4 diets were formulated to be marginally low in crude protein (183 g/kg). Challenged groups were inoculated with a solution containing *E. maxima, E. acervulina*, and *E. tenella* oocysts on d 15. *Eimeria* depressed (*P* < 0.01) growth performance and nutrient utilization. Supplemental protease improved (*P* < 0.05) body weight gain and feed intake in the prechallenge phase (d 0–15) but had no effect during the infection period (d 15–21). There was no interaction between infection and feed supplementation for nutrient utilization. The supplementations of either PRO or XOS alone increased (*P* < 0.01) total tract retention of Ca and tended (*P* < 0.1) to improve total tract retention of N, P, AME, and AMEn. *Eimeria* decreased (*P* < 0.05) expressions of GLUT2, GLUT5, PepT1, ATP2B1, CaSR, Calbidin D28K, NPT2, and ZnT1 but increased (*P* < 0.01) expression of GLUT1. XOS supplementation increased (*P* < 0.05) ATP2B1 expression. Protease decreased (*P* < 0.05) isobutyrate concentration in unchallenged treatments but not in challenged treatments. *Eimeria* decreased (*P* < 0.01) cecal saccharolytic SCFAs acetate and propionate but increased (*P* < 0.01) branched-chain fatty acid isovalerate. The supplementation of PRO + XYL or XOS increased (*P* < 0.05) cecal butyrate or decreased cecal isobutyrate concentrations, respectively. PRO + XYL and XOS decreased cecal protein levels in unchallenged birds but not challenged ones. *Eimeria* challenge significantly (*P* < 0.05) decreased the microbial richness (Observed features) and diversity (Shannon index and phylogenetic diversity) and changed the microbial composition by reducing the abundance of certain bacteria, such as *Ruminococcus torques*, and increasing the abundance of others, such as *Anaerostipes*. In contrast, none of the additives had any significant effect on the cecal microbial composition. In conclusion, PRO or XOS supplementation individually improved nutrient utilization. All the additives decreased the cecal content of branched-chain fatty acids, consistent with decreased cecal N concentration, although the effects were more pronounced in unchallenged birds. In addition, none of the feed additives impacted the *Eimeria*-induced microbial perturbation.

## INTRODUCTION

Coccidiosis, caused by *Eimeria* spp., is a protozoan parasitic disease that extensively occurs in poultry (Chapman et al., 2013). According to an annual survey conducted by the United States Animal Health Association, coccidiosis has been ranked as the number one concerning disease in the broiler chicken industry for a decade (The United States Animal Health Association, 2020). *Eimeria* colonizes birds’ intestinal epithelial cells, multiplies, and eventually destroys the cells, resulting in cell necrosis, gut lesions, hemorrhage, and depressed production in both layer and broiler chickens. Therefore, coccidiosis results in an annual global economic loss of up to $14 billion in the broiler industry due to production losses and expenses on prevention or treatment (Blake et al., 2020).

It has also been shown that coccidiosis causes a drastic shift in the intestinal microbiome (Macdonald et al., 2017; Campos et al., 2022). Coccidiosis is one of the most important predisposing factors of *Clostridium perfringens*-induced necrotic enteritis, suggesting a strong association between *Eimeria* disease and the growth of *Clostridium perfringens* (Timbermont et al., 2011). However, because of the complexity and volatility of the intestinal bacteria, the effect of *Eimeria* infection on other microflora remains unclear (Moore, 2016). On the other hand, supplementing the feed with additives capable of modulating intestinal microbiota may be a nutritional approach to improve gut health and promote recovery from coccidiosis in infected broiler chickens.

The effects of exogenous protease or carbohydrates on improving growth performance in coccidiosis-infected chickens have been demonstrated (Jackson et al., 2003; Peek et al., 2009). For example, supplemental enzymes resulted in a higher body weight gain (**WG**) and a thicker gut mucus layer in infected chickens (Peek et al., 2009). The potential of exogenous enzymes and prebiotics to partly alleviate the harmful effects of *Eimeria* may be partially explained based on their capacity to beneficially alter the gut microbial profile and thus improve gut health. For example, protease can enhance protein and amino acid digestibility, especially in diets with low protein or inherently low amino acid digestibility (Angel et al., 2011a; Cowieson et al., 2017b). Therefore, protease may reduce the relative proportion of undigested protein reaching the ceca, which can otherwise promote proliferation of pathogenic bacteria.

As an emerging prebiotic from agricultural products, xylo-oligosaccharides (**XOS**) are able to influence the gut microflora by being utilized as growth substrates by beneficial bacteria (Singh et al., 2021). This is supported by our previous observation that XOS supplementation preferentially enhanced carbohydrates fermentation while inhibiting protein fermentation in the hindgut, possibly indicative of selective utilization by beneficial microorganisms (Lin et al., 2022). Carbohydrates fermentation produces beneficial metabolites such as short-chain fatty acids (**SCFAs**) instead of harmful metabolites of protein fermentation such as ammonia. In addition, with enzymes as a mainstay in the broiler industry, the combined application of carbohydrases and protease may provide benefits beyond enhanced nutrient utilization efficiency. The synergistic effect of protease and carbohydrase has been shown to increase the hydrolysis of complex carbohydrate substrates and nutrient utilization including N and AME (Olukosi et al., 2015). Furthermore, enzyme and prebiotic supplementation can induce changes in the content of carbohydrates and proteins in the digesta, for example, reducing the level of N and increasing oligosaccharides in the hindgut, leading to a favorable environment for proliferation of intestinal microbial community that can partly ameliorate parasite-induced intestinal microbial disorders.

The current experiment investigated the potential and mechanisms of exogenous protease, xylanase, and xylo-oligosaccharides supplementation to mitigate the *Eimeria* challenge's deleterious effects on growth performance and nutrient utilization, and microbiota in broiler chickens. The low-protein diets used in the current experiment were used to maximize the effect of protease based on results from our previous study (Lin and Olukosi, 2021a).

## MATERIALS AND METHODS

### Birds, Diets, Experimental Design, and *Eimeria* Challenge

This study was approved by the Institutional Animal Care and Use Committee of the University of Georgia, Athens, GA and conducted at the Poultry Science Research Complex, University of Georgia.

Three hundred ninety-two 0-day-old Cobb 500 (off-sex) male broiler chicks were used in the 21-day experiment to study the potential of dietary protease, protease combined with xylanase, or XOS to partly alleviate *Eimeria*-induced harmful effects in broiler chickens receiving a low-protein diet. Low-protein (183 g/kg) corn-soybean meal diets (Table 1) were formulated with phytase supplemented at 500 FTU/kg (Quantum Blue, AB Vista, Marlborough, UK; 5,000 FTU/g). Birds were allocated to 8 treatments in a 4 × 2 factorial arrangement, and each of the 8 treatments had 7 replicate cages, 7 birds per replicate cage. One of the factors was the additive supplemented: basal diet without additive supplementation (**Con**), a basal diet with 0.2 g/kg protease (**PRO**) (DSM, Pendergrass, GA), 0.2 g/kg protease combined with 0.1 g/kg xylanase (AB Vista, Marlborough, UK) (**PRO + XYL**), and 0.5 g/kg XOS (AIDP Inc., City of Industry, CA) on top. The second factor was the *Eimeria* challenge (with or without). The serine protease used in this study was the fermented product of the sporulation-deficient *Bacillus licheniformis* strain (Kalmendal and Tauson, 2012). The β-(1-4)-endo-xylanase was produced from the fermentation of genetically modified *Trichoderma reesei* (Lin and Olukosi, 2021b). The food-grade XOS was obtained from nongenetically modified corn and was previously characterized (Yang et al., 2015; Silva et al., 2020). The water-based *Eimeria* spp. solution used to challenge the birds consisted of 12,500 oocysts/mL of *E. maxima*, 12,500 oocysts/mL of *E. tenella*, and 62,500 oocysts/mL of *E. acervuline*, and was used to stimulate a mild infection (Teng et al., 2020).

**Table 1:** Ingredients and analyzed compositions (g/kg) of the experimental control diets.

Items	Basal
Corn	702
Soybean meal	250
Soybean oil	10
Titanium dioxide	5
Dicalcium phosphate	9
Limestone	11
Lysine	3.8
Methionine	1.6
Threonine	1.4
NaHCO_3_	0.8
Salt	3.2
Vitamin premix^1^	2.5
Trace minerals premix^2^	2.5
Phytase	0.1
Total	1,000
Calculated nutrients and energy, g/kg	
Crude protein	183
ME, kcal/kg	3,087
Ca	7.4
Total P	5.4
Available P^3^	3.0
Met	4.6
Cys	3.0
Met + Cys	7.7
Lys	12.2
His	5.0
Trp	2.2
Thr	8.4
Arg	11.8
Analyzed composition of experimental diets, g/kg	
Dry matter	879
Crude protein	181
Calcium	7.12
Total phosphorus	5.03
Neutral detergent fiber	7.73
Acid detergent fiber	4.16
Hemicellulose	3.57

1 Vitamin A, 5,484 IU; vitamin D3, 2,643 ICU; vitamin E, 11 IU; menadione sodium bisulfite, 4.38 mg; riboflavin, 5.49 mg, D-pantothenic acid, 11 mg; niacin, 44.1 mg, choline chloride, 771 mg; vitamin B12, 13.2 µg; biotin, 55.2 µg; thiamine mononitrate, 2.2 mg; folic acid, 990 µg; pyridoxine hydrochloride, 3.3 mg.

2 Iodine, 1.11 mg; manganese, 66.06 mg; copper, 4.44 mg; iron, 44.1 mg; zinc, 44.1 mg; selenium, 300 µg.

3 Available P level included the matrix value for the phytase.

On d 15, birds in challenge treatments were orally gavaged with 1 mL/bird mixed-species *Eimeria* oocysts solution, whereas birds in unchallenged treatments received 1 mL/bird water as a placebo. Ten extra birds were raised (receiving basal diet) in a separate cage for blank blood samples as the standard reference for the subsequent gut permeability test.

### Growth Performance, Intestinal Permeability, and Lesion Scoring

Birds (per cage) and feed were weighed on d 0, 15, and 21 for calculation of body WG, feed intake (**FI**), and gain: feed for both the prechallenge (d 0–15) and challenge phases (d 15–21).

The intestinal permeability test was performed 5-day postinfection (**dpi**) on d 20, using a modification of (Baxter et al., 2017) protocol. Briefly, 1 bird was randomly selected from each challenged cage and the unchallenged Con treatment and administrated with 1 mL of freshly prepared 2.2 mg/mL fluorescein isothiocyanate dextran (**FITC-d**, MW 4,000; Sigma-Aldrich, St. Louis, MO) solution. Blank blood samples from extra birds were collected to dilute FITC-d for the standard curve preparation. Two hours after oral administration of FITC-d to the birds, the birds were euthanized, and blood samples were collected from the heart. Clotted blood was centrifuged at 1,000 × *g* for 12 min. The serum was collected and measured by spectrophotometer (Spectramax M5, Molecular Devices, San Jose, CA) at the wavelength of 485 nm excitation and 528 nm emission. All the blood processing procedures were done in a dark room.

At d 21, 3 birds per cage were used to score intestinal lesions based on a 0 to 4 scale grading (no lesion to severe lesion) according to the method previously described (Johnson and Reid, 1970). The upper (duodenum), middle (jejunum and ileum), and ceca sections of the intestine were scored separately.

### Collection of Samples

The excreta were collected at d 20 (5 dpi) and subsequently dried (75°C) in an oven drier for 3 d. The dried excreta were used for the total tract retention measurements of nitrogen, calcium, and phosphorus. Ileal digesta samples were collected from 5 birds per cage at d 21 (6 dpi), and the samples were then oven-dried at 75°C for 3 d for ileal digestibility measurements. Cecal contents were collected from 3 birds per cage on d 21 and stored at −20°C for later SCFAs, protein concentration measurement, and microbiota analysis. Jejunal mucosa was collected from 2 birds per cage, immediately snap-frozen in liquid N, and stored at −80°C before further gene expression analysis.

### Oocyst Shedding

Excreta at d 21 (6 dpi) were collected quantitatively from cages for oocyst shedding measurement as described by Conway and Mckenzie (2007). After thorough mixing, approximately 5 g excreta samples from each cage were weighed and diluted with water in a 1:99 ratio. After vortexing, 5 mL of diluted samples were mixed with 45 mL of saturated salt solution in a centrifuge tube. After appropriate vortexing, the samples were loaded in a McMaster chamber and observed under a microscope. The total oocyst shed was counted and standardized as oocysts per gram of excreta.

### Quantitative Real-Time PCR and 16S rRNA Gene Sequencing Analysis

Gene expression of intestinal nutrient transporters in jejunum mucosa was analyzed by Quantitative real-time PCR. Samples were homogenized with a QiAzol lysis reagent (QIAGEN, Hilden, Germany), and total RNA was extracted according to the manufacturer's instructions. Extracted RNA was converted to cDNA in a 20 μL reaction volume by high-capacity cDNA reverse transcription kit (Thermo Fisher Scientific, Waltham, MA) after quantity measurement in BioTek Synergy HTX spectrophotometer (Agilent, Santa Clara, CA) and diluted to equal concentration. The quantitative reverse-transcriptase polymerase chain reaction was performed in Step One Plus real-time PCR system (Thermo Fisher Scientific, Waltham, MA) with reaction master mix iTaq Universal SYBR Green Supermix (Bio-Rad, Hercules, CA). Samples were run in duplicate, and the $2^{-\Delta \Delta Ct}$ method (Livak and Schmittgen, 2001) was applied to analyze the results. All the primers used in the experiment are listed in Table 2.

**Table 2:** Forward and reverse primers for quantitative PCR analysis.

Gene symbol	Accession number	Full name	Function	Forward primer	Reverse primer
18S	MG967540.1	18S ribosomal RNA	Housekeeping gene	AGCCTGCGGCTTAATTTGAC	CAACTAAGAACGGCCATGCA
Beta-actin	NM_205518.1	Beta-actin	Housekeeping gene	CAACACAGTGCTGTCTGGTGGTA	ATCGTACTCCTGCTTGCTGATCC
GAPGH	NM_204305.2	Glyceraldehyde-3-phosphate dehydrogenase	Housekeeping gene	GAGGGTAGTGAAGGCTGCTG	CCACAACACGGTTGCTGTAT
PepT1 (SLC15A1)	KF366603.1	Peptide transporter-1	Peptide transporter	CCCCTGAGGAGGATCACTGTT	CAAAAGAGCAGCAGCAACGA
GLUT1 (SLC2A1)	NM_205209.1	Glucose transporter-1	Glucose transporter	CTTTGTCAACCGCTTTGG	CAGAATACAGGCCGATGAT
GLUT2 (SLC2A2)	XM_010716927.3	Glucose transporter-2	Glucose transporter	TCATTGTAGCTGAGCTGTT	CGAAGACAACGAACACATAC
GLUT5 (SLC2A5)	XR_005855627.1	Glucose transporter-5	Glucose transporter	TTGCTGGCTTTGGGTTGTG	GGAGGTTGAGGGCCAAAGTC
SGLT1 (SLC5A1)	NM_001293240.1	Sodium glucose transporter-1	Glucose transporter	GCCGTGGCCAGGGCTTA	CAATAACCTGATCTGTGCACCAGT
SGLT4 (SLC5A9)	XM_040678521.2	Sodium glucose transporter-4	Glucose transporter	ATACCCAAGGTAATAGTCCCAAAC	TGGGTCCCTGAACAAATGAAA
ATP2B1	XM_046906440.1	ATPase plasma membrane Ca^2+^ transporting 1	Ca^2+^ transporter	TTAATGCCCGGAAAATTCAC	TCCACCAAACTGCACGATAA
CASR	XM_040661543.2	Calcium sensing receptor	Calcium receptor	GCCAATCTGCTGGGACTCTT	CTGATGCTCGTCATTGGGGA
Calbidin D28k	AH003256.2	Calbindin-D28K	Calcium transporter	TTTGATGCAAACAATGATGGA	TTTTGCACACATTTTGACACC
NPT2	AY389468.2	Sodium phosphate cotransporter	Phosphate cotransporter	GGAAGCATTGCTGCGGATT	GCACCCTCCTGTTCTGCATT
ZnT1 (SLC22A18)	XM_040673965.1	Zinc transporter-1	Zinc transporter	TCCGGGAGTAATGGAAATCTTC	AATCAGGAACAAACCTATGGGAAA

DNA was extracted from cecal content by QIAamp PowerFecal Pro DNA Kit (QIAGEN, Germantown, MD) following the manufacturer's instructions. Following DNA extraction, samples were sent to LC Sciences, LLC (Houston, TX) for library preparation and 16S rRNA gene sequencing using an Illumina MiSeq instrument (Illumina Inc., San Diego, CA). Forward primer S-D-Bact-0341-b-S-17 (5′-CCTACGGGNGGCWGCAG-3′) and reverse primer S-D-Bact-0785-a-A-21 (5′-GACTACHVGGGTATCTAATCC-3′) were used for PCR libraries. The DNA sequence data were analyzed by Qiime2 following previously described procedures (Akerele et al., 2022).

### Chemical Analysis

Oven-dried diets, excreta, and ileal digesta were ground (0.5 mm) to measure dry matter (**DM**) (AOAC Method 934.01), nitrogen (**N**), gross energy (**GE**), and titanium. A combustion nitrogen analyzer (LECO, St. Joseph, MI) measured N content in diets, ileal digesta, cecal content, and excreta (AOAC Method 968.06). The gross energy and mineral profile of diets and excreta were measured by the Central Analytical Laboratory, University of Arkansas. The gross energy was measured by an isoperibol bomb calorimeter (Model 6200, Parr Instruments, Moline, IL) with benzoic acid as the calibration standard, and minerals were measured by Spectro Analytical instruments (Arcos OES ICP, Kleve, Germany) (AOAC 968.08-1969). Titanium concentration in the samples was determined according to the method of Short et al. (1996). Cecal SCFAs composition was analyzed by gas chromatography using the methodology described by Lourenco et al., 2020. Briefly, around 1 g cecal content sample was diluted in deionized water in a 1:3 ratio in 15 mL tubes. The solution was vortexed, and 1.5 mL of the mix was centrifuged at 10,000 × *g* for 10 min. The supernatant was collected and mixed well with 25% (wt/vol) meta-phosphoric acid solution. After overnight frozen, the mixture was thawed, centrifuged and the supernatant was mixed with ethyl acetate in a ratio of 1: 2. After vortexed and settled for 5 min, the mixture's top layer was transferred to a glass vial and analyzed by gas chromatography.

### Calculations and Statistical Analysis

The index method was used to calculate total tract retention and apparent ileal digestibility of energy, DM, crude protein, and minerals using the following equation:

$$\text{Digestibility}=100\;\times \;\left\{1-\left[\left(\frac{C_{i}}{C_{o}}\right)\times \left(\frac{N_{o}}{N_{i}}\right)\right]\right\}$$

where *C*_i_ is the concentration of titanium in the diet, *N*_i_ is the nutrient content in the diet, *C*_o_ is the concentration of titanium in excreta or digesta, and *N*_o_ is the nutrient content in excreta or ileal digesta.

The following 2 equations calculated apparent metabolizable energy and AMEn:

$$\text{AME}=GE_{i}-\left[\left(\frac{C_{i}}{C_{o}}\right)\times GE_{o}\right]$$

$$\text{AMEn}=\text{AME}-\left(8.22\;\times \frac{\text{NR}}{\text{DMI}}\right)$$

where GE_i_ is the gross energy of the diet and GE_o_ is the gross energy of the excreta. NR is the retained nitrogen (g) and DMI is the dry matter intake (kg).

The data were analyzed by the mixed model procedure of JMP (SAS Institute Inc., Cary, NC) as appropriate for a randomized complete block design and a factorial treatment arrangement. The comparison of treatments, except for lesion scores and microbiota, was subjected to 2-way ANOVA. The 2 factors were the 2 levels of the *Eimeria* challenge and 4 types of supplementations. Tukey's honestly significant difference test was used to separate means if there is a significant interaction. Kruskal-Wallis nonparametric statistical method was used for intestinal lesion score, alpha diversity indices, relative bacterial richness, and comparisons of microbial composition between treatments. Statistical significance was set at *P* ≤ 0.05, and trends were set at *P* < 0.10.

## RESULTS

### Growth Performance and Nutrient Utilization

In the prechallenge phase, birds receiving diets with protease had greater (*P* < 0.01) WG and FI. In the challenge phase, the *Eimeria* challenge significantly (*P* < 0.01) decreased WG, FI, and gain:feed, whereas PRO and XOS tended (*P* = 0.08) to increase FI (Table 3).

**Table 3:** Growth performance response of broiler chickens receiving protease, protease plus xylanase, or prebiotic oligosaccharides in diets formulated to be marginally lower in crude protein and challenged, or not, with mixed *Eimeria* spp.

			Prechallenge phase (d 0–15)				Challenge phase (d 15–21)		
Treatment	*Eimeria*	Supplementation	WG, g	FI, g	Gain: feed, g/kg		WG, g	FI, g	Gain: feed, g/kg
1	−	Con	399	581	687		396	467	848
2	−	PRO	426	614	694		479	509	939
3	−	PRO + XYL	420	593	708		419	490	857
4	−	XOS	400	565	707		452	497	909
5	+	Con	403	588	685		289	408	707
6	+	PRO	435	615	708		285	410	696
7	+	PRO + XYL	413	575	718		306	408	744
8	+	XOS	387	581	667		292	410	711
Pooled SEM			11.3	10.7	15.0		18.6	8.3	33.7
Means for main effect of *Eimeria* challenge									
	−						437	491	888
	+						293	409	714
Pooled SEM							9.8	4.4	16.9
*P* values for main effect of *Eimeria* challenge							<0.001	<0.001	<0.001
Means for main effect of supplementations									
		Con	401^b^	585^b^		686	342	437	778
		PRO	431^a^	614^a^		701	381	459	817
		PRO + XYL	416^ab^	584^b^		713	362	449	801
		XOS	393^b^	573^b^		686	372	454	810
Pooled SEM			7.8	7.5		10.7	24.1	12.8	34.0
*P* values for main effect of supplementations			0.007	0.002		0.246	0.188	0.082	0.375
*P* values for interactions							0.075	0.134	0.230

*n* = 7 replicates for the simple effects; *n* = 28 replicates for the main effects of *Eimeria* challenge; *n* = 14 replicates for the main effects of additives supplementation.

WG: weight gain; FI: feed intake; Con: no supplementation; PRO: protease; PRO + XYL: protease and xylanase; XOS: xylo-oligosaccharides.

Means along a column with different superscripts (a, b) are significantly different (*P* < 0.05).

*Eimeria* challenge significantly (*P* < 0.01) lowered ileal DM and N digestibility by 24.6 and 32.6%, respectively (Table 4). Birds fed with PRO had the highest DM (66.2%) and N (67.9%) digestibility, greater (*P* < 0.05) than the birds receiving PRO + XYL, which had the lowest DM (55.3%) and N (54.9%) digestibility. Broiler chickens in challenged treatments showed depressed (*P* < 0.01) AME, AMEn, and total tract retention of N, Ca, and P. Birds receiving the diet supplemented with XOS or PRO had the highest (*P* < 0.05) total tract retention of N, AME, AMEn, Ca, and P. In contrast, birds in PRO + XYL group had the lowest (*P* < 0.05) total tract retention for all of the nutrients and energy.

**Table 4:** Total tract nutrient retention and ileal digestibility responses of 21-day-old broiler chickens receiving protease, protease plus xylanase, or prebiotic oligosaccharides in diets formulated to be marginally lower in crude protein and challenged, or not, with mixed *Eimeria* spp.

				Ileal digestibility (%)		Total tract retention				
Treatment	*Eimeria*		Supplementation	DM	Nitrogen	Nitrogen	AME, kcal/kg	AMEn, kcal/kg	Ca, %	P, %
1	−		Con	72.6	74.3	65.8	2944	2864	42.6	46.4
2	−		PRO	74.1	78.5	59.7	2964	2884	51.7	50.3
3	−		PRO + XYL	69.3	73.5	66.5	2801	2709	38.8	40.6
4	−		XOS	74.9	79.8	67.2	3013	2933	50.6	49.8
5	+		Con	43.7	39.1	41.3	2062	1923	22.2	22.1
6	+		PRO	58.3	57.3	37.4	2212	2094	31.0	31.3
7	+		PRO + XYL	41.3	36.3	50.8	1933	1790	23.8	24.3
8	+		XOS	48.9	43.1	50.1	2257	2135	37.5	31.4
Pooled SEM				4.4	3.0	2.9	81.7	87.9	2.8	3.5
Means for main effect of *Eimeria* challenge										
	−			72.7	76.5	64.8	2931	2847	45.9	46.7
	+			48.1	43.9	44.9	2116	1985	28.6	27.3
Pooled SEM				1.7	2.3	1.6	43.6	46.9	1.7	1.8
*P* values for main effect of *Eimeria* challenge				<0.001	<0.001	<0.001	<0.001	<0.001	<0.001	<0.001
Means for main effect of supplementations										
			Con	58.1^ab^	56.7^ab^	53.5^ab^	2503^ab^	2393^ab^	32.4^b^	34.2^ab^
			PRO	66.2^a^	67.9^a^	58.7^a^	2588^a^	2489^a^	41.3^a^	40.8^a^
			PRO + XYL	55.3^b^	54.9^b^	48.6^b^	2367^b^	2250^b^	31.3^b^	32.5^b^
			XOS	61.9^ab^	61.4^ab^	58.7^a^	2635^a^	2534^a^	44.1^a^	40.6^a^
Pooled SEM				4.1	5.5	3.4	126	134	3.1	3.6
*P* values for main effect of supplementations				0.006	0.018	<0.001	<0.001	0.009	<0.001	0.004
*P* values for interactions				0.147	0.202	0.349	0.755	0.729	0.393	0.461

*n* = 7 replicates for the simple effect; *n* = 28 replicates for the main effects of *Eimeria* challenge; *n* = 14 replicates for the main effects of each supplementation.

Con: no supplementation; PRO: protease; PRO + XYL: protease and xylanase; XOS: xylo-oligosaccharides.

Means along a column with different superscripts (a, b) are significantly different (*P* < 0.05).

### Intestinal Permeability, Lesion Scores, and Oocyst Shedding

The gastrointestinal permeability response on d 20 (5 dpi) showed that birds challenged with mixed *Eimeria* species had numerically higher serum FITC-d levels, indicating greater intestinal leakage due to *Eimeria* spp. invasion (data shown in supplementary file). Supplementations had no significant effect on intestinal permeability. The intestinal lesion scores showed that *E. acervuline, E. maxima*, and *E. tenella* produced intestinal lesions in the upper intestine, middle intestine, and ceca, respectively (data shown in supplementary file). Supplementations had no significant effect on intestinal lesion scores. Oocyst shedding was observed in all *Eimeria*-challenged birds, whereas supplementations had no impact on oocyst numbers (data shown in supplementary file).

### Gene Expression of Nutrients Transporters

The significant *Eimeria* × supplementations interaction (*P* < 0.05) for GLUT2 showed that supplementations of PRO + XYL or XOS tended (*P* < 0.10) to upwardly express GLUT2 in challenged treatments but had the opposite effect in unchallenged treatments (Table 5). In addition, the *Eimeria* challenge produced a downward (*P* < 0.05) expression of glucose transporter GLUT5, peptide transporter PepT1, Ca transporter ATP2B1 and calbidin D28K, calcium receptor CaSR, phosphate cotransporter NPT2, and zinc transporter ZnT1, whereas it produced an upward expression of glucose transporter GLUT1. Supplemental XOS produced upward (*P* < 0.05) expression of ATP2B1.

**Table 5:** Gene expression of nutrient transporters in the jejunum of 21-day-old broiler chickens at 6-day postchallenge after receiving diets supplemented with protease, protease plus xylanase, or prebiotic oligosaccharides in diets formulated to be marginally lower in crude protein and challenged, or not with mixed *Eimeria* spp.

Treatment	*Eimeria*	Supplementation	GLUT1	GLUT2	GLUT5	SGLT1	SGLT4	PepT1	ATP2B1	CaSR	Calbidin D28K	NPT2	ZnT1
1	−	Con	1.000	1.000^ab^	1.000	1.000	1.000	1.000	1.000	1.000	1.000	1.000	1.000
2	−	PRO	1.518	1.226^a^	0.941	1.361	0.844	1.183	1.319	2.658	1.340	0.963	1.011
3	−	PRO + XYL	0.754	0.884^abc^	0.669	0.859	1.864	0.599	1.322	1.506	1.309	1.029	0.738
4	−	XOS	1.019	0.848^abc^	0.730	0.930	1.018	1.071	1.863	0.992	1.493	1.655	1.049
5	+	Con	2.922	0.499^bc^	0.487	1.101	1.020	0.546	0.616	0.555	0.549	0.506	0.494
6	+	PRO	4.363	0.379^c^	0.331	0.778	1.247	0.507	0.633	0.633	0.290	0.473	0.544
7	+	PRO + XYL	3.504	0.681^abc^	0.466	0.802	1.095	0.664	0.554	0.650	0.228	0.449	0.459
8	+	XOS	2.491	0.582^bc^	0.367	0.725	0.992	0.873	0.909	0.743	0.549	0.565	0.704
Pooled SEM			0.421	0.044	0.044	0.032	0.106	0.051	0.056	0.265	0.076	0.096	0.040
Means for main effect of *Eimeria* challenge													
	−		1.073	0.989	0.835	1.038	1.181	0.963	1.376	1.539	1.286	1.162	0.949
	+		3.320	0.535	0.413	0.851	1.088	0.648	0.678	0.645	0.404	0.498	0.550
Pooled SEM													
*P* values for main effect of *Eimeria* challenge			<0.001	<0.001	0.007	0.125	0.700	0.040	0.003	0.003	<0.001	0.014	0.011
Pooled SEM													
Means for main effect of supplementations													
		Con	1.961	0.749	0.743	1.051	1.010	0.773	0.808^b^	0.777	0.775	0.753	0.747
		PRO	2.940	0.802	0.636	1.070	1.480	0.845	0.976^ab^	1.646	0.815	0.718	0.778
		PRO + XYL	2.129	0.782	0.567	0.830	1.045	0.632	0.938^ab^	1.078	0.769	0.739	0.598
		XOS	1.755	0.715	0.549	0.827	1.005	0.972	1.386^a^	0.868	1.021	1.110	0.877
Pooled SEM			0.232	0.064	0.063	0.064	0.103	0.073	0.088	0.118	0.086	0.097	0.073
*P* values for main effect of supplementations			0.181	0.805	0.656	0.207	0.279	0.344	0.025	0.272	0.660	0.197	0.357
*P* values for interactions			0.578	0.045	0.659	0.139	0.214	0.283	0.454	0.245	0.492	0.538	0.836

*n* = 7 replicates for the simple effect; *n* = 28 replicates for the main effects of *Eimeria* challenge; *n* = 14 replicates for the main effects of each supplementation.

Con: no supplementation; PRO: protease; PRO + XYL: protease and xylanase; XOS: xylo-oligosaccharides.

Means along a column with different superscripts (a–c) are significantly different (*P* < 0.05).

### Cecal Short-Chain Fatty Acids Profile and Protein Concentration

The *Eimeria* × supplementations interaction for cecal isobutyrate concentration showed that the supplementation of all the additives decreased (*P* < 0.05) the concentration of isobutyrate in unchallenged treatments but not in the challenged treatments (Table 6). The interaction for cecal isovalerate concentration showed that the supplementation of XOS decreased (*P* < 0.05) the concentration of isovalerate in unchallenged treatments but not in the challenged treatments. The profile of SCFAs indicated that birds challenged with *Eimeria* spp. had lower (*P* < 0.01) concentrations of saccharolytic SCFAs acetate and propionate but higher (*P* < 0.01) concentrations of butyrate, valerate, branched-chain fatty acids (**BCFAs**) isobutyrate, and isovalerate. All the other additives tended to increase the saccharolytic SCFAs acetate and total SCFAs. In addition, PRO + XYL significantly increased (*P* < 0.05) the cecal content of saccharolytic SCFAs butyrate, and XOS supplementation significantly decreased (*P* < 0.05) the level of isovalerate. Table 7 shows the total cecal protein concentration in treatments. *Eimeria* × supplementations interaction (*P* < 0.05) showed that PRO + XYL and XOS decreased the total protein level in cecal content in unchallenged birds but not in challenged birds.

**Table 6:** Cecal short-chain fatty acid profile (mM) in 21-day-old broiler chickens at 6-day postchallenge after receiving diets supplemented with protease, protease plus xylanase, or prebiotic oligosaccharides in diets formulated to be marginally lower in crude protein and challenged, or not with mixed *Eimeria* spp.

Treatment	*Eimeria*	Supplementation	Acetate	Propionate	Isobutyrate	Butyrate	Isovalerate	Valerate	Total SCFA
1	−	Con	70.4	4.32	0.785^a^	10.7	0.912^abc^	1.072	88.2
2	−	PRO	84.5	3.78	0.323^c^	17.7	0.342^bcd^	0.911	107.5
3	−	PRO + XYL	88.1	4.27	0.394^bc^	17.3	0.369^cd^	1.063	111.5
4	−	XOS	89.4	4.29	0.283^c^	16.9	0.300^d^	0.960	112.2
5	+	Con	65.4	3.15	0.798^a^	19.9	1.113^a^	1.194	91.5
6	+	PRO	68.9	3.10	1.047^a^	23.5	1.363^a^	1.546	99.5
7	+	PRO + XYL	69.0	3.23	0.734^ab^	25.3	1.056^a^	1.442	100.8
8	+	XOS	66.7	3.28	0.750^ab^	19.4	0.962^ab^	1.249	92.3
Pooled SEM			3.96	0.432	0.085	2.545	0.133	0.128	6.23
Means for main effect of *Eimeria* challenge									
	−		83.1	4.16	0.446	15.7	0.481	1.002	104.9
	+		67.5	3.19	0.833	22.0	1.123	1.358	96.0
Pooled SEM			2.05	0.206	0.051	1.31	0.074	0.064	3.27
*P* values			<0.001	<0.001	<0.001	<0.001	<0.001	<0.001	0.059
Means for main effect of supplementations									
		Con	67.9	3.73	0.792	15.3^b^	1.012	1.133	89.9
		PRO	78.5	3.75	0.564	21.3^ab^	0.712	1.253	106.2
		PRO + XYL	76.7	3.44	0.695	20.6^a^	0.852	1.229	103.5
		XOS	78.1	3.78	0.517	18.2^ab^	0.631	1.105	102.3
Pooled SEM			2.05	0.206	0.051	1.31	0.074	0.064	3.27
*P* values for main effect of supplementations			0.068	0.851	0.011	0.033	0.026	0.496	0.059
*P* values for interactions			0.237	0.954	0.002	0.427	0.025	0.163	0.337

*n* = 7 replicates for the simple effect; *n* = 28 replicates for the main effects of *Eimeria* challenge; *n* = 14 replicates for the main effects of each supplementation.

Con: no supplementation; PRO: protease; PRO + XYL: protease and xylanase; XOS: xylo-oligosaccharides.

Means along a column with different superscripts (a–c) are significantly different (*P* < 0.05).

**Table 7:** Cecal protein concentration (μg/mg) in 21-day-old broiler chickens at 6-day postchallenge after receiving diets supplemented with protease, protease plus xylanase, or prebiotic oligosaccharides in diets formulated to be marginally lower in crude protein and challenged, or not with mixed *Eimeria* spp.

Treatment	*Eimeria*	Supplementation	Protein concentration
1	−	Con	51.3^ab^
2	−	PRO	48.9^bcd^
3	−	PRO + XYL	47.4^d^
4	−	XOS	47.7^cd^
5	+	Con	50.5^abcd^
6	+	PRO	52.9^a^
7	+	PRO + XYL	51.4^abc^
8	+	XOS	50.3^abcd^
Pooled SEM			1.04
Means for main effect of *Eimeria* challenge			
	−		48.8
	+		51.3
Pooled SEM			0.544
*P* values			0.008
Means for main effect of supplementations			
		Con	50.9
		PRO	50.9
		PRO + XYL	49.4
		XOS	49.0
Pooled SEM			0.86
*P* values for main effect of supplementations			0.028
*P* values for interactions			0.017

*n* = 7 replicates for the simple effect; *n* = 28 replicates for the main effects of *Eimeria* challenge; *n* = 14 replicates for the main effects of each supplementation.

Con: no supplementation; PRO: protease; PRO + XYL: protease and xylanase; XOS: xylo-oligosaccharides.

Means along a column with different superscripts (a–d) are significantly different (*P* < 0.05).

### Cecal Microbial Profile

Microbial richness and diversity for the birds in different treatments are shown in Table 8. Supplementation of the additives had no effects on microbial richness or diversity indexes. The number of observed features was significantly lower (*P* < 0.01) in challenged birds, indicating a lower richness of the microbiota in the challenged treatments. The challenge decreased both the Shannon diversity index and Faith's phylogenetic diversity index (*P* < 0.05), demonstrating the effect of coccidiosis in decreasing microbial diversity.

**Table 8:** Effect of *Eimeria* infection on richness and diversity in cecal samples collected on 6-day postchallenge in 21-day old broiler chickens after receiving diets supplemented with protease, protease plus xylanase, or prebiotic oligosaccharides.

Treatment	*Eimeria*	Supplementation	Observed ASV^1^	Faith's phylogenetic diversity	Shannon index
1	−	Con	196	10.76	6.04
2	−	PRO	185	9.85	6.11
3	−	PRO + XYL	177	9.62	6.09
4	−	XOS	195	10.46	6.04
5	+	Con	149	9.06	5.58
6	+	PRO	161	9.39	5.83
7	+	PRO + XYL	166	9.85	5.79
8	+	XOS	137	8.50	5.21
Pooled SEM		11.9	0.51	0.27	
*P* values for treatments	<0.01	0.079	0.344		
Means for main effect of *Eimeria* challenge					
	−		188	10.17	6.07
	+		153	9.20	5.60
Pooled SEM		5.9	0.26	0.13	
*P* values for main effect of *Eimeria* challenge	<0.001	0.011	<0.001		
Means for main effect of supplementations					
		Con	172	9.91	9.91
		PRO	173	9.62	9.62
		PRO + XYL	171	9.73	9.73
		XOS	166	9.48	9.48
Pooled SEM		10.0	0.40	0.20	
*P* values for main effect of supplementations	0.983	0.851	0.863		

Diets were formulated to be marginally lower in crude protein, and birds were challenged or not with mixed *Eimeria* spp.

1 Observed amplicon sequence variant.

**Figure 1:**
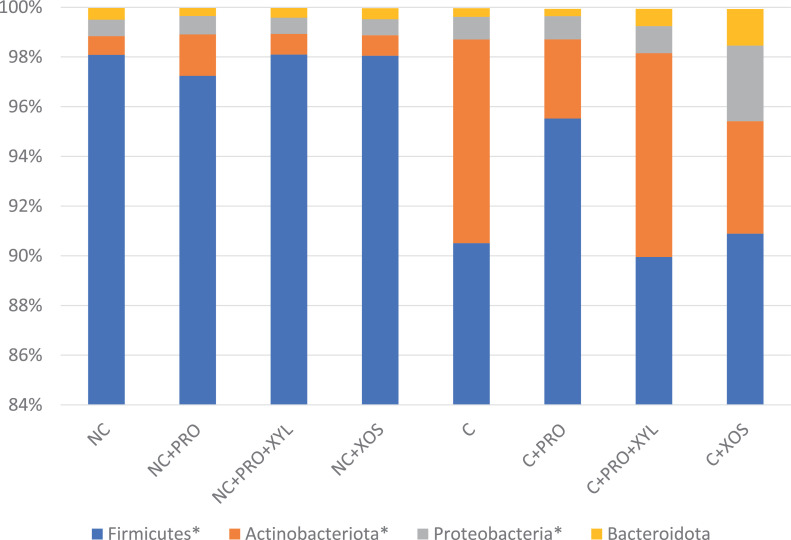
Bar chart showing relative abundance of bacterial phyla in each treatment (6 dpi). *N* = 6. NC, unchallenged-no supplementation treatment; NC + PRO, challenged and supplemented with protease treatment; NC + PRO + XYL, challenged and supplemented with protease and xylanase treatment; C + XOS, challenged and supplemented with xylo-oligosaccharides treatment; C, challenged-no supplementation treatment; C + PRO, challenged and supplemented with protease treatment; C + PRO + XYL, challenged and supplemented with protease and xylanase treatment; C + XOS, challenged and supplemented with xylo-oligosaccharides treatment. Only phyla with relative abundances ≥1% in at least one sample type are shown. *Indicates a *P* value ≤0.05 for the contrast: unchallenged vs. challenge.

None of the supplemented additives showed any effects on microbial composition at the phylum level. On the other hand, the microbial composition was significantly influenced by *Eimeria* infection (Figure 1). *Eimeria* infection significantly (*P* < 0.01) decreased the abundance of Firmicutes, which comprised the greatest percentage of all phyla in the nonchallenged birds. On the other hand, the composition of Actinobacteria and Proteobacteria was significantly (*P* < 0.05) higher in the challenged treatments.

Bacterial genera with significantly different abundances due to challenge are shown in Table 9. *Eimeria* challenge decreased (*P* < 0.05) the abundance of *Butyricicoccus, Klebsiella, Marvinbryantia, Pseudoflavonifractor, Romboutsia*, and *Shuttleworthia* but increased (*P* < 0.05) the relative abundance of *Anaerostipes, ASF356, Bifidobacterium, CHKCI002, Clostridioides, Clostridium sensu stricto 1, Enterococcus, Escherichia-Shigella, Lactobacillus*, and *Sellimonas*. At the species level, *Eimeria* infection increased (*P* < 0.05) the composition of *Clostridium perfringens* which belongs to the genus *Clostridium sensu stricto 1* (shown in supplemental tables). Dietary supplementation with the additives showed no effect on genus abundance except for PRO increasing (*P* < 0.05) the relative abundance of the *Eubacterium coprostanoligenes* group (shown in supplemental tables).

**Table 9:** Bacterial genera with significantly different abundances (%) in cecal samples collected on 6-day postchallenge of 21-day-old broiler chickens challenged, or not with mixed *Eimeria* spp.

Bacterial genus	Unchallenged	Challenged	*P* value
*Ruminococcus*_*torques*_group (f: Lachnospiraceae, p: Firmicutes)	13.943	8.378	<0.01
*Anaerostipes* (f: Lachnospiraceae, p: Firmicutes)	1.850	6.206	<0.01
*ASF356* (f: Lachnospiraceae, p: Firmicutes)	0.079	0.226	<0.01
*Bifidobacterium* (f: Bifidobacteriaceae, p: Actinobacteriota)	0.196	4.687	<0.01
*Butyricicoccus* (f: Butyricicoccaceae, p: Firmicutes)	1.766	0.954	<0.01
*CHKCI002* (f: Eggerthellaceae, p: Actinobacteriota)	0.775	1.304	<0.01
*Clostridioides* (f: Peptostreptococcaceae, p: Firmicutes)	0.002	0.014	0.048
*Clostridium sensu stricto 1* (f: Clostridiaceae, p: Firmicutes)	0.006	0.204	0.046
*Enterococcus* (f: Enterococcaceae, p: Firmicutes)	0.041	0.175	<0.01
*Escherichia-Shigella* (f: Enterobacteriaceae, p: Proteobacteria)	0.386	1.356	<0.01
*Klebsiella* (f: Enterobacteriaceae, p: Proteobacteria)	0.163	0.054	<0.01
*Lactobacillus* (f: Lactobacillaceae, p: Firmicutes)	2.204	11.555	<0.01
*Marvinbryantia* (f: Lachnospiraceae, p: Firmicutes)	0.069	0.000	<0.01
*Pseudoflavonifractor* (f: Oscillospiraceae, p: Firmicutes)	0.064	0.040	0.022
*Romboutsia* (f: Peptostreptococcaceae, p: Firmicutes)	0.273	0.014	<0.01
*Sellimonas* (f: Lachnospiraceae, p: Firmicutes)	1.223	2.212	<0.01
*Shuttleworthia* (f: Lachnospiraceae, p: Firmicutes)	0.618	0.339	<0.01

## DISCUSSION

In this study, the *Eimeria* challenge resulted in depression of growth performance and nutrient utilization, as evidenced by a 33% reduction in body WG and a 24.6% reduction of ileal N digestibility. The above, combined with the result of gut permeability, lesion score, and oocyst shedding, indicate a successful mild infection. Among the feed additives, only PRO supplementation improved growth performance in the prechallenge phase and tended to increase FI in the challenge phase. The application of exogenous protease in livestock is less common compared with phytase or carbohydrases. On the other hand, proteases have been promising, because they helped improve growth performance and nutrient utilization in broilers (Angel et al., 2011b; Cowieson et al., 2017a). Regarding nutrient utilization, individual supplementation of PRO and XOS improved Ca total tract retention, emphasizing the beneficial effects of enzyme and prebiotics on mineral bioavailability. In a similar vein, the ability of XOS to enhance mineral absorption has been demonstrated in our previous study (Lin et al., 2022). Increasing mineral availability is a common occurrence with the prebiotic application, which can be explained by the effect on the microbiome and alterations of pH and SCFAs profile (Whisner and Castillo, 2018). Hindgut bacteria ferment prebiotics such as XOS and produce SCFAs, thereby creating a lower luminal pH environment, which may limit potential complexation of phytate and minerals. The free mineral has greater solubility and bioavailability than those complexed with phytate under conditions of higher pH. Acidity-induced increase in free-Ca flux and absorption stimulate Ca-transporter gene expression as shown previously (Scholz-Ahrens et al., 2001) and in the current study.

*Eimeria* infection impacts the gene expression of many nutrient transporters in the enterocytes, including glucose, protein, and mineral transporters. In agreement with previous studies (Su et al., 2014, 2015; Teng et al., 2021), the current experiment showed that the parasite challenge produced downward expressions of all the tested genes, except for GLUT1 which was upwardly expressed by 3-fold. The increased expression of GLUT1 may be partly explained by its basolateral location in the intestine. It was hypothesized that the nutrient transporter alteration under *Eimeria* infection achieves the outcome of epithelial apoptosis. By decreasing the expression of brush border transporters and increasing basolateral membrane transporters, apoptosis follows from nutritional depletion (Paris and Wong, 2013; Su et al., 2014). The upward expression of basolateral ZnT1 will likely result in Zn accumulation, accelerating apoptosis because of metal poisoning (Su et al., 2015). Regarding the effects of feed additives supplementation, XOS produced an upward expression of ATP2B1, as mentioned previously, consistent with the finding of improved Ca total tract retention in the current study. In addition, the interaction between feed additives and infection showed that PRO + XYL and XOS supplementation reversed the *Eimeria*-induced GLUT2 downward expression. The downregulated GLUT2 by *Eimeria* infection was previously reported (Su et al., 2014; Teng et al., 2021). However, the mechanism by which the infection downregulated GLUT2 and how the additives reverses the effect is not known.

Most SCFAs, including acetate, butyrate, propionate, and valerate are fermentation products from nondigestible carbohydrates. Although less abundant in quantity, some SCFAs known as BCFAs, such as isobutyrate and isovalerate, are produced from the fermentation of protein or amino acid substrates (Tan et al., 2014). Accordingly, the profile of SCFAs reflects the condition of intestinal fermentation patterns and microbial activity. Short-chain fatty acids play important roles in maintaining gut health and enterocyte functions. For instance, butyrate is the primary energy source for enterocytes, contributing 60 to 70% of their energy requirements (Wächtershäuser and Stein, 2000; Tan et al., 2014).

The profile of SCFAs is dynamic and can be altered by factors such as diet, feed additives, and disease (Topping and Clifton, 2001; Van Immerseel et al., 2004; Koh et al., 2016). In the current study, consistent with previous works (Lin and Olukosi, 2021b; Lin et al., 2022), SCFAs concentration increased or tended to increase with the additives supplementation. On the contrary, the cecal content of analyzed BCFAs such as isobutyrate and isovalerate decreased with the additives supplementation. It can be reasoned that the exogenous enzymes and prebiotics supplemented preferentially promoted carbohydrates, rather than proteins, fermentation in the hindgut by increasing the activity of carbohydrate-hydrolyzing bacteria. It is worth noting that, in the current study, enzymes and prebiotics also modulated the cecal environment by reducing the cecal N level, which could be one of their modes of action. By decreasing available nitrogenous substrates, exogenous enzymes and prebiotics limit the action of protein-fermenting bacteria, thereby reducing the cecal level of BCFAs.

*Eimeria* infection was previously reported to lower the concentration of SCFAs and increase the concentration of BCFAs (Lin and Olukosi, 2021b; Lin et al., 2022), but not always (Wils-Plotz et al., 2013; Craig et al., 2020; Choi et al., 2021). In the current experiment, the *Eimeria* challenge increased BCFAs and decreased SCFAs except for butyrate and valerate, which showed an increase. In line with the findings from an experiment measuring the SCFAs level at both 6 and 9 dpi (Choi et al., 2021), the SFCA profile following a challenge is quite dynamic, starting with a significant reduction in concentration followed by an acute, but transient increase, and then a gradual return to normal, prechallenge level, as the birds recover. As shown in the current study and previous coccidiosis research, the transient improvement of SCFAs is likely to start with the concentration of butyrate (Hilliar et al., 2020). The acute increase in cecal butyrate is accompanied by a sudden increase of *Bifidobacterium*, symptomatic of the rapid and turbulent changes following the infection. It also may imply a vigorous comeback for beneficial bacteria when the microbial ecosystem returns to normal after an acute infection.

The impacts of coccidiosis infection on the microbiota reported in different studies varied broadly, showing the complexity and intractable instability of microbial changes following the disease onset. For instance, the different dosages of *Eimeria* infection, time points after infection, or intestinal section collected all produced significant variabilities in microbial profile (Macdonald et al., 2017; Campos et al., 2022). The consistently confirmed bacteria species linked with *Eimeria* infection is necrotic enteritis-inducing *Clostridium perfringens* which was also elevated by the infection in the current experiment.

In agreement with the literature (Madlala et al., 2021), the coccidiosis challenge in the current study reduced the microbial richness and diversity, which also likely explains the quantitatively lower total cecal SCFAs production in challenged birds, indicating a negative impact of the challenge on the microbial ecosystem. Moreover, in more severe cases of the challenge, most studies reported a decrease in beneficial microbial taxa. In the current study, however, a higher abundance of *Bifidobacterium* and *Lactobacillus* was observed in challenged treatments, as also reported in other mild infection cases (Macdonald et al., 2017; Campos et al., 2022). One possible explanation is that, as an acute infectious disease, *Eimeria* causes significant, but transient, damage to the chicken gastrointestinal tract and the microbial ecology, but one from which the birds recover after a short while. Simultaneously, the beneficial bacteria seem to overcompensate to re-establish the microbial balance in the ecosystem, leading to a transient domination by certain beneficial bacteria with abnormally high values of SCFAs during the recovery stage (Choi et al., 2021). The elevated *Lactobacillus* and *Bifidobacterium* can modulate the innate immune system and stimulate immune factors, promoting recovery from the infection (Dalloul et al., 2005). For example, in the current study, the level of butyrate was positively correlated (*P* < 0.01) with the abundance of *Bifidobacterium* (data not shown). It is worth noting that challenged birds had a higher abundance of *Clostridium perfringens* and the whole *Clostridium sensu stricto 1* genus, highlighting that *Eimeria* infection has a strong association with necrotic enteritis. The decreased *Ruminococcus, Romboutsia*, and *Shuttleworthia* and the increased pathogens *Escherichia-Shigella* were also reported in the previous studies, related to opportunistic outbreaks due to intestinal infection (Macdonald et al., 2017; Chen et al., 2020; Memon et al., 2022). Although positive effects of enzymes or prebiotics on mitigating the influence of coccidiosis on the intestinal microbiota has been previously reported (Bortoluzzi et al., 2019), we did not observe such effects in the current study.

In conclusion, PRO or XOS individually supplemented were superior to combination of PRO + XYL in promoting growth performance in prechallenge phase and at improving nutrient utilization in *Eimeria*-infected birds. All the additives produced favorable SCFAs profile by regulating hindgut fermentation, consistent with decreased cecal N concentration, indicating that improving SCFA profile is one of the modes of action by which the studied additives supported gut health in broiler chickens. Nevertheless, the additives preferentially driving carbohydrates fermentation was more apparent in nonchallenged birds. In addition, the supplemented additives had no significant impact on *Eimeria*-induced intestinal microbiota perturbation.

## Appendix Supplementary materials

Image, application 1

Image, application 2

Image, application 3

## Footnotes

Supplementary material associated with this article can be found in the online version at doi:10.1016/j.psj.2023.102789.
