# Supplementary material for: The effects of protease, xylanase, and xylo-oligosaccharides on growth performance, nutrient utilization, short-chain fatty acids, and microbiota in Eimeria-challenged broiler chickens fed low-protein diet
Source: Poult Sci. 2023 May 19;102(8):102789. doi: 10.1016/j.psj.2023.102789 (PMC10404748; doi:10.1016/j.psj.2023.102789)
Supplement: Supplementary file 1 [file mmc1.pptx]

## Slide 1
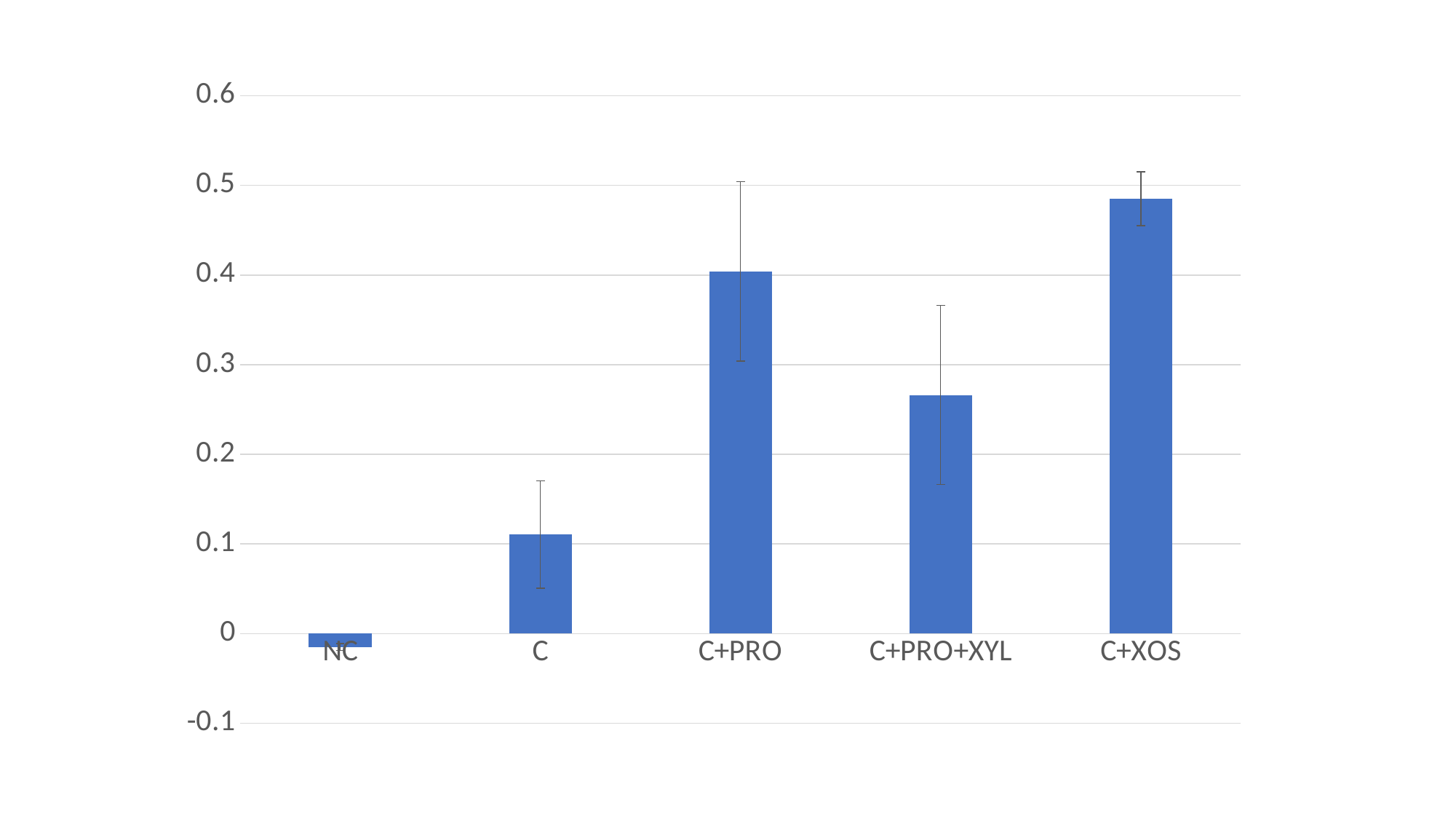

### Chart
| Category | |
|---|---|
| NC | -0.015 |
| C | 0.1105317 |
| C+PRO | 0.404 |
| C+PRO+XYL | 0.2662033682142857 |
| C+XOS | 0.485 |

## Slide 2
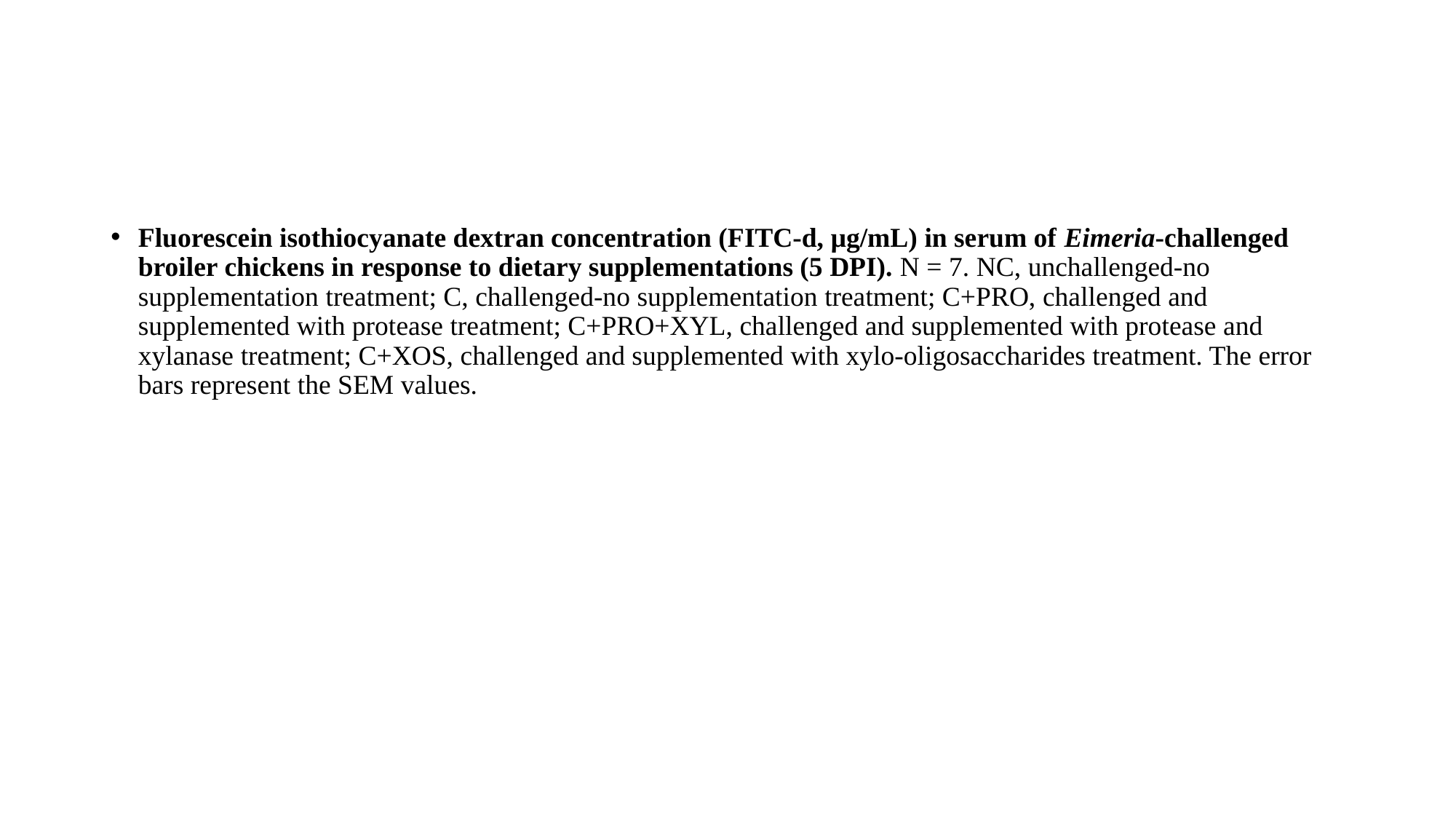

#
Fluorescein isothiocyanate dextran concentration (FITC-d, µg/mL) in serum of Eimeria-challenged broiler chickens in response to dietary supplementations (5 DPI). N = 7. NC, unchallenged-no supplementation treatment; C, challenged-no supplementation treatment; C+PRO, challenged and supplemented with protease treatment; C+PRO+XYL, challenged and supplemented with protease and xylanase treatment; C+XOS, challenged and supplemented with xylo-oligosaccharides treatment. The error bars represent the SEM values.
