# Supplementary material for: The effects of protease, xylanase, and xylo-oligosaccharides on growth performance, nutrient utilization, short-chain fatty acids, and microbiota in Eimeria-challenged broiler chickens fed low-protein diet
Source: Poult Sci. 2023 May 19;102(8):102789. doi: 10.1016/j.psj.2023.102789 (PMC10404748; doi:10.1016/j.psj.2023.102789)
Supplement: Supplementary file 2 [file mmc2.pptx]

## Slide 1
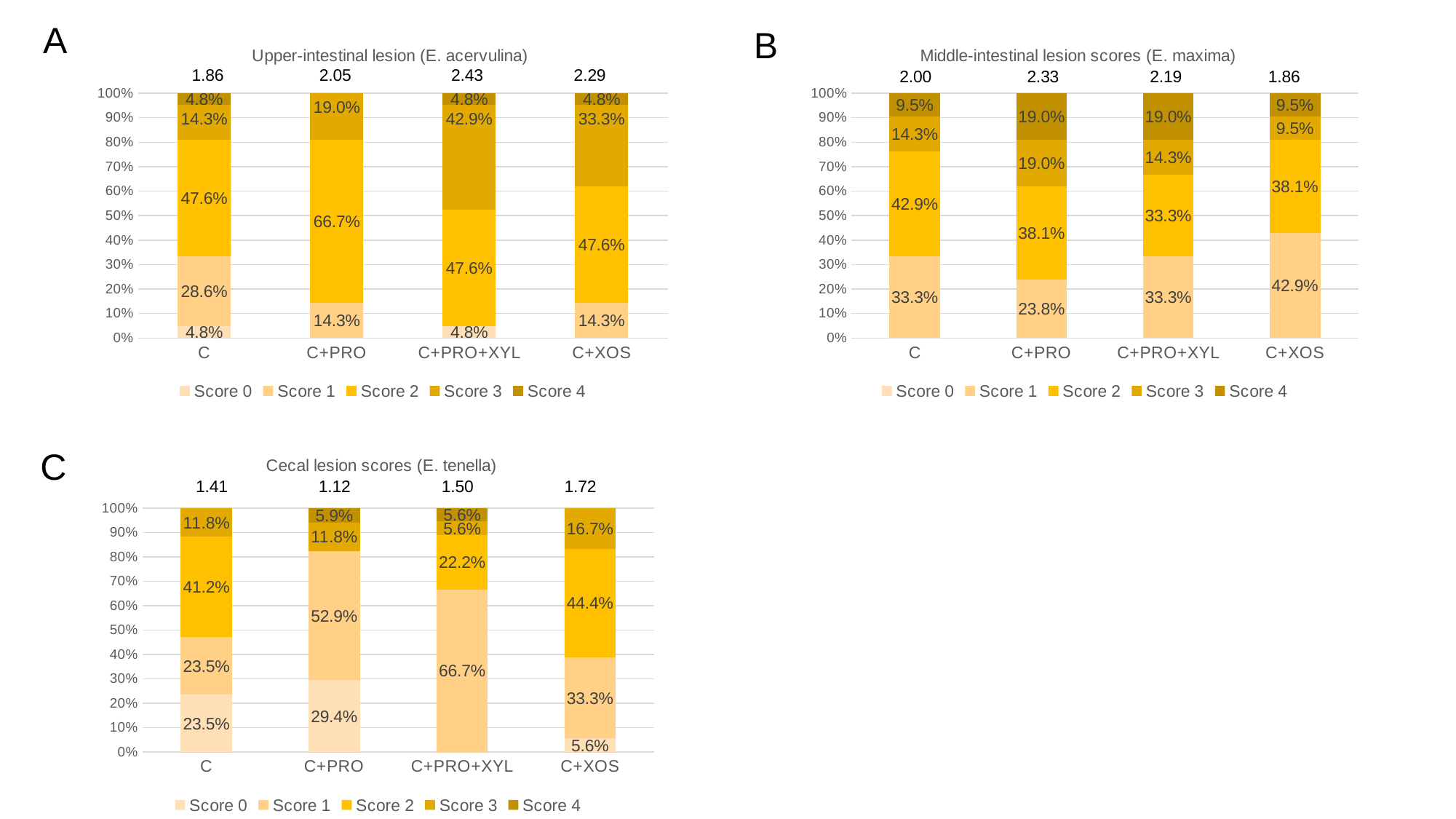

A
B
### Chart: Upper-intestinal lesion (E. acervulina)
| Category | | | | | |
|---|---|---|---|---|---|
| C | 0.047619047619047616 | 0.2857142857142857 | 0.47619047619047616 | 0.14285714285714285 | 0.047619047619047616 |
| C+PRO | 0.0 | 0.14285714285714285 | 0.6666666666666666 | 0.19047619047619047 | 0.0 |
| C+PRO+XYL | 0.047619047619047616 | 0.0 | 0.47619047619047616 | 0.42857142857142855 | 0.047619047619047616 |
| C+XOS | 0.0 | 0.14285714285714285 | 0.47619047619047616 | 0.3333333333333333 | 0.047619047619047616 |
### Chart: Middle-intestinal lesion scores (E. maxima)
| Category | | | | | |
|---|---|---|---|---|---|
| C | 0.0 | 0.3333333333333333 | 0.42857142857142855 | 0.14285714285714285 | 0.09523809523809523 |
| C+PRO | 0.0 | 0.23809523809523808 | 0.38095238095238093 | 0.19047619047619047 | 0.19047619047619047 |
| C+PRO+XYL | 0.0 | 0.3333333333333333 | 0.3333333333333333 | 0.14285714285714285 | 0.19047619047619047 |
| C+XOS | 0.0 | 0.42857142857142855 | 0.38095238095238093 | 0.09523809523809523 | 0.09523809523809523 |C
### Chart: Cecal lesion scores (E. tenella)
| Category | | | | | |
|---|---|---|---|---|---|
| C | 0.23529411764705882 | 0.23529411764705882 | 0.4117647058823529 | 0.11764705882352941 | 0.0 |
| C+PRO | 0.29411764705882354 | 0.5294117647058824 | 0.0 | 0.11764705882352941 | 0.058823529411764705 |
| C+PRO+XYL | 0.0 | 0.6666666666666666 | 0.2222222222222222 | 0.05555555555555555 | 0.05555555555555555 |
| C+XOS | 0.05555555555555555 | 0.3333333333333333 | 0.4444444444444444 | 0.16666666666666666 | 0.0 |

## Slide 2
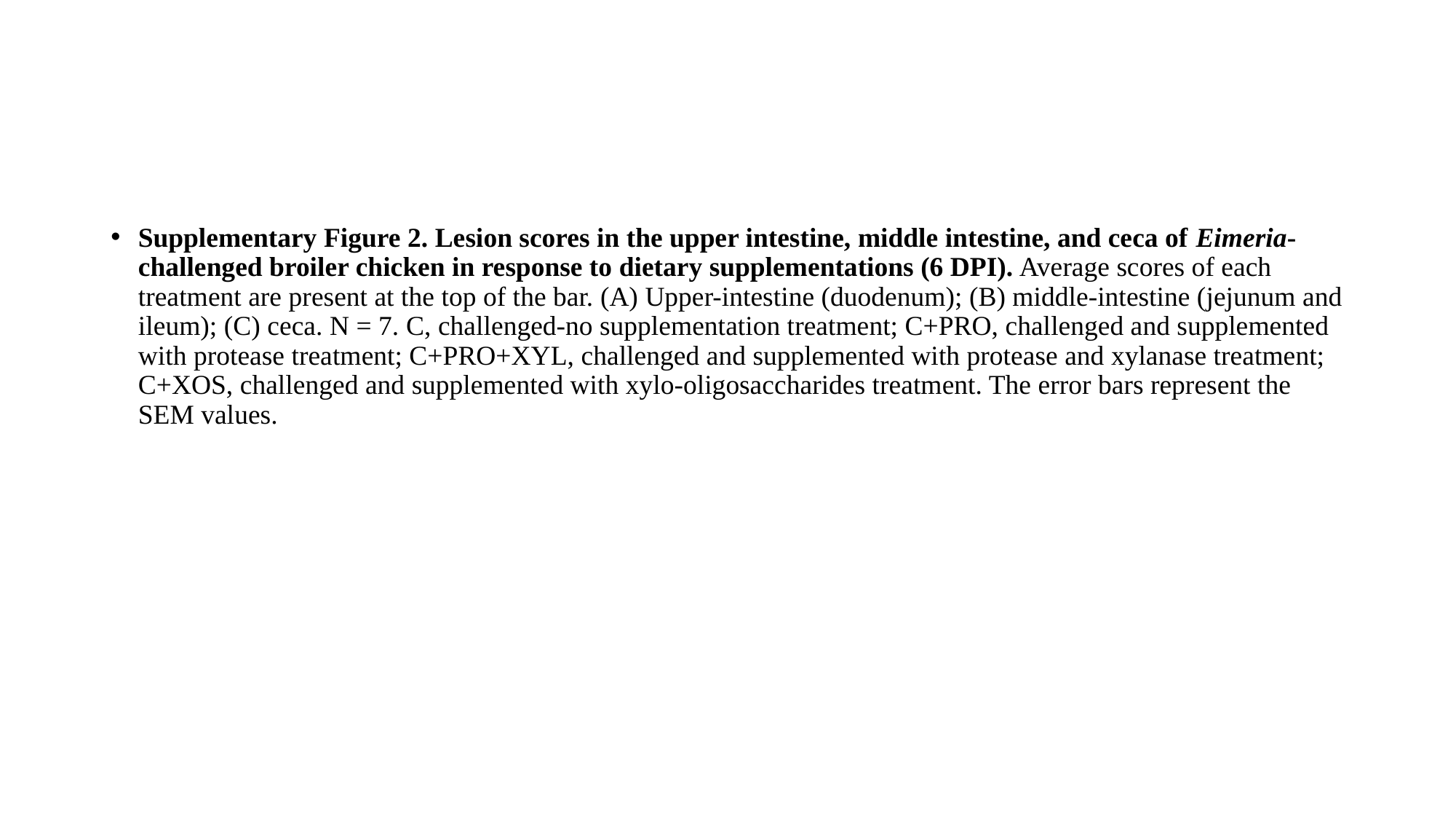

#
Supplementary Figure 2. Lesion scores in the upper intestine, middle intestine, and ceca of Eimeria-challenged broiler chicken in response to dietary supplementations (6 DPI). Average scores of each treatment are present at the top of the bar. (A) Upper-intestine (duodenum); (B) middle-intestine (jejunum and ileum); (C) ceca. N = 7. C, challenged-no supplementation treatment; C+PRO, challenged and supplemented with protease treatment; C+PRO+XYL, challenged and supplemented with protease and xylanase treatment; C+XOS, challenged and supplemented with xylo-oligosaccharides treatment. The error bars represent the SEM values.
