# Supplementary material for: The effects of protease, xylanase, and xylo-oligosaccharides on growth performance, nutrient utilization, short-chain fatty acids, and microbiota in Eimeria-challenged broiler chickens fed low-protein diet
Source: Poult Sci. 2023 May 19;102(8):102789. doi: 10.1016/j.psj.2023.102789 (PMC10404748; doi:10.1016/j.psj.2023.102789)
Supplement: Supplementary file 3 [file mmc3.pptx]

## Slide 1
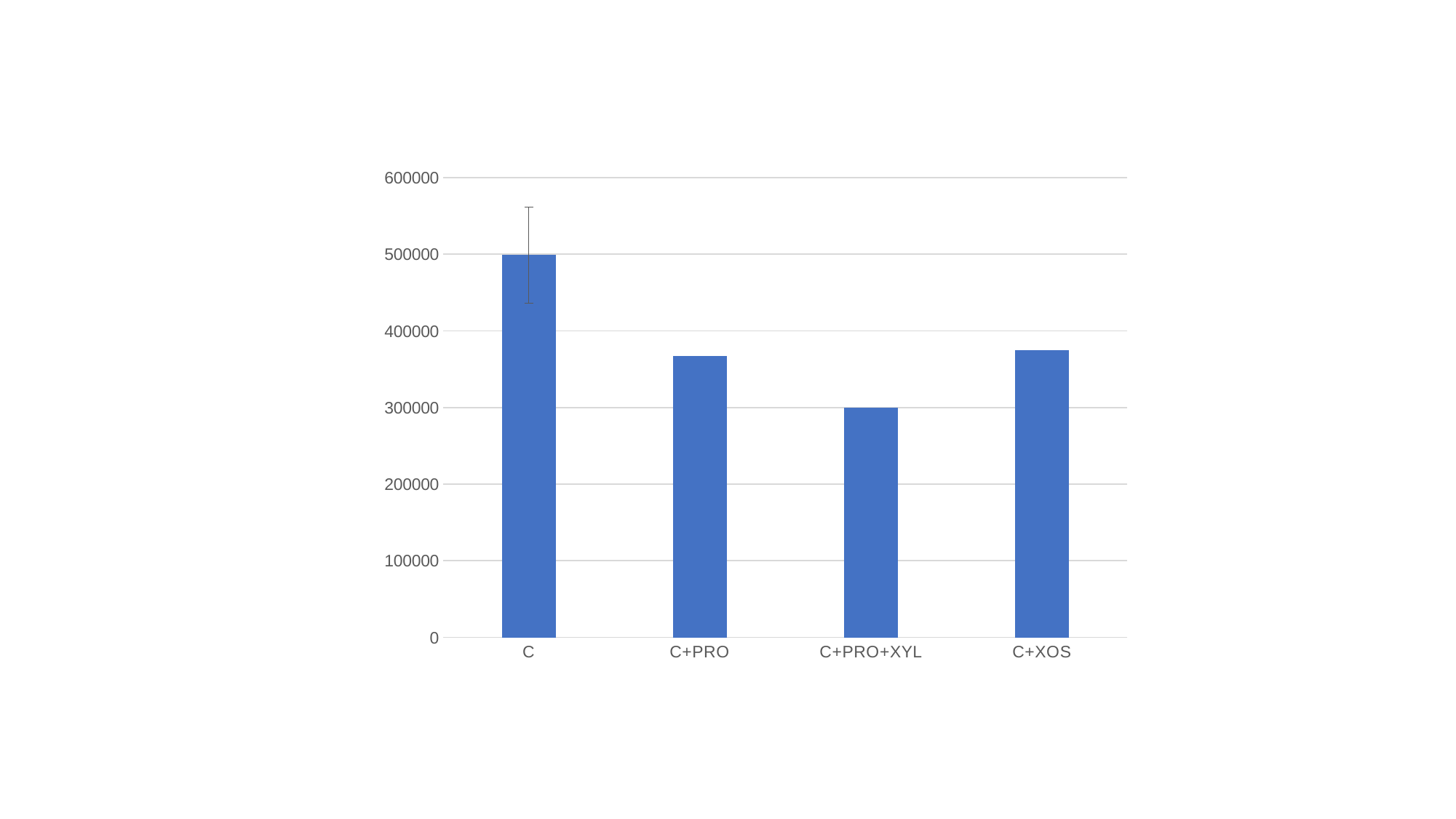

### Chart
| Category | |
|---|---|
| C | 498942.8571428571 |
| C+PRO | 367504.7619047619 |
| C+PRO+XYL | 299457.14285714284 |
| C+XOS | 375314.2857142857 |

## Slide 2
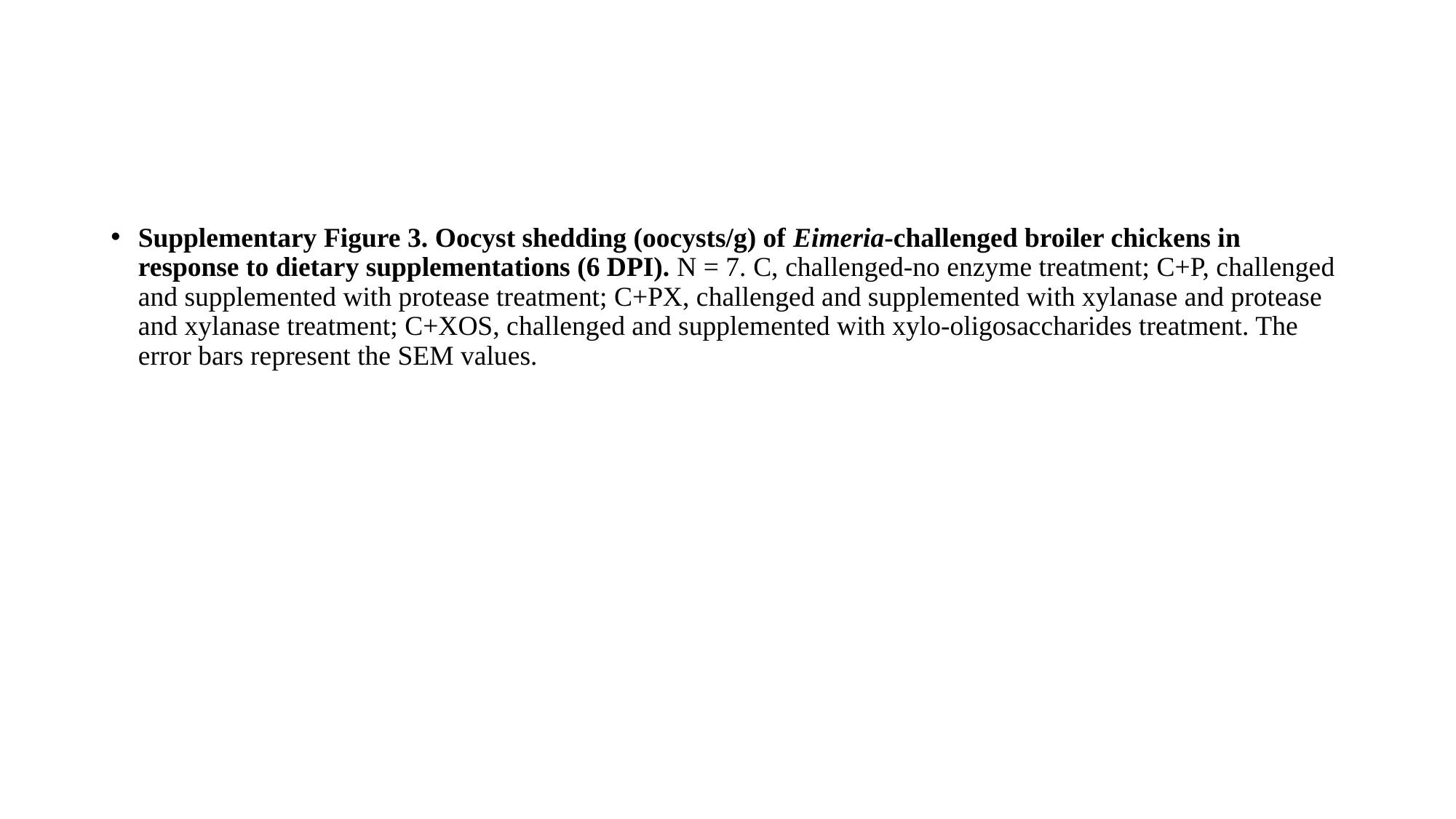

#
Supplementary Figure 3. Oocyst shedding (oocysts/g) of Eimeria-challenged broiler chickens in response to dietary supplementations (6 DPI). N = 7. C, challenged-no enzyme treatment; C+P, challenged and supplemented with protease treatment; C+PX, challenged and supplemented with xylanase and protease and xylanase treatment; C+XOS, challenged and supplemented with xylo-oligosaccharides treatment. The error bars represent the SEM values.
